# Indirect Genetic Effects and the Dynamics of Social Interactions

**DOI:** 10.1371/journal.pone.0126907

**Published:** 2015-05-18

**Authors:** Barbora Trubenová, Sebastian Novak, Reinmar Hager

**Affiliations:** 1 Institute of Science and Technology Austria (IST Austria), 3400 Klosterneuburg, Austria; 2 Computational and Evolutionary Biology, Faculty of Life Sciences, University of Manchester, Manchester M13 9PT, United Kingdom; University of Sheffield, UNITED KINGDOM

## Abstract

**Background:**

Indirect genetic effects (IGEs) occur when genes expressed in one individual alter the expression of traits in social partners. Previous studies focused on the evolutionary consequences and evolutionary dynamics of IGEs, using equilibrium solutions to predict phenotypes in subsequent generations. However, whether or not such steady states may be reached may depend on the dynamics of interactions themselves.

**Results:**

In our study, we focus on the dynamics of social interactions and indirect genetic effects and investigate how they modify phenotypes over time. Unlike previous IGE studies, we do not analyse evolutionary dynamics; rather we consider within-individual phenotypic changes, also referred to as phenotypic plasticity. We analyse iterative interactions, when individuals interact in a series of discontinuous events, and investigate the stability of steady state solutions and the dependence on model parameters, such as population size, strength, and the nature of interactions. We show that for interactions where a feedback loop occurs, the possible parameter space of interaction strength is fairly limited, affecting the evolutionary consequences of IGEs. We discuss the implications of our results for current IGE model predictions and their limitations.

## Introduction

When individuals form social groups (e.g. families or herds) their phenotypes are affected by the social environment they experience. For example, in mammalian families mothers influence their offspring’s development through provisioning and other maternal behaviours [1–3]. The effects of an individual’s genes on the phenotype of social partners are referred to as indirect genetic effects (IGEs) [4–11] or associative effects [12–14].

The importance of IGEs for trait variation and their consequences for the evolution of social behaviours has been shown in a number of studies. IGEs can speed up or slow down the evolution of traits and change the direction of evolution from what would be expected in the absence of IGEs [4, 15–17]. Moreover, IGEs may also lead to differences in the direction between phenotypic and genotypic response to selection [18]. Furthermore, IGEs can create non-random associations of phenotypes and thus allow evolutionary responses to social selection to occur. In addition, Bijma and Wade [19] showed that IGEs can lead to selection at the group level.

All of the aforementioned studies focused on evolutionary consequences and evolutionary dynamics of IGEs, investigating changes of phenotypes in subsequent generations. However, they ignored the dynamics of interactions, i.e. how individual phenotypes change during their lifetime or during a single interaction, and how these changes are influenced by the nature of these interactions. The implicit assumption of IGE models is that individual phenotypes at the point of selection are at an equilibrium point (also referred to as stationary point, fixed point or steady state; e.g. [20]), thus not changing in time, defined by a set of stationary parameters such as genotypes and interaction strengths. For instance, if aggressive behaviour and displays of dominance lead to individuals assuming a stable position within the hierarchy, IGE models usually consider only the hierarchical structure, or dominance as a trait of interest, not the gradual changes in aggressive behaviour leading to such a state. This often appears a reasonable assumption given that phenotypic changes occur on a different time scale than evolutionary changes. Moreover, it seems reasonable to assume that most interactions among individuals lead in the end to a state where phenotypes do not change dramatically over time. However, equilibrium points may be stable or unstable—a system slightly disturbed from the stable equilibrium will return to the same point, but even a small perturbation of the system from an unstable equilibrium will lead to a departure of the system from this equilibrium [21, 22]. In real populations some fluctuations in the social environment will inevitably occur, moving the system away from the equilibrium state. For instance, the arrival of a new group member may decrease the time other members spend being vigilant [23–25], or increase aggressive behaviour [26].

While behavioural dynamics and its evolutionary implications have been thoroughly investigated by game theory [27–31], this is not commonly done in quantitative genetics studies. The importance of considering behavioural dynamics in evolutionary studies has been highlighted only recently [32–34]. A new two-tiered framework has been developed by Akçay et al. [33], who modelled social interactions at the behavioural time scale (first tier), and in the subsequent step then considered evolutionary dynamics based on the phenotypes and fitness resulting from the interactions (second tier).

While Akçay and Van Cleve [35] have recently shown that it is possible to map between the two-tiered model and the IGE framework, to our knowledge, no study has investigated stability of solution used by IGE models. Therefore, in this study we analyse equilibrium solutions used by traditional IGE models and their stability. We show that in the context of IGEs it is crucial to consider the dynamics of interactions and the stability of assumed equilibrium states upon which evolutionary dynamics are based, for instance by following the two tiered approach suggested by [33, 35, 36]

In this study, we focus on an example of simple iterative interactions, when individuals modify their phenotypes at each iteration, dependent on the phenotypes of others they experienced in the previous encounter. We do not analyze evolutionary dynamics, instead, we investigate how individual phenotypes change over time (phenotypic plasticity) and how this development is influenced by the nature of interactions, strength of interaction and the number of interacting individuals. We illustrate these dependences using agent based simulations. Finally, we discuss the implications of our results for current IGE model predictions and their limitations.

## Model

**Fig 1:**
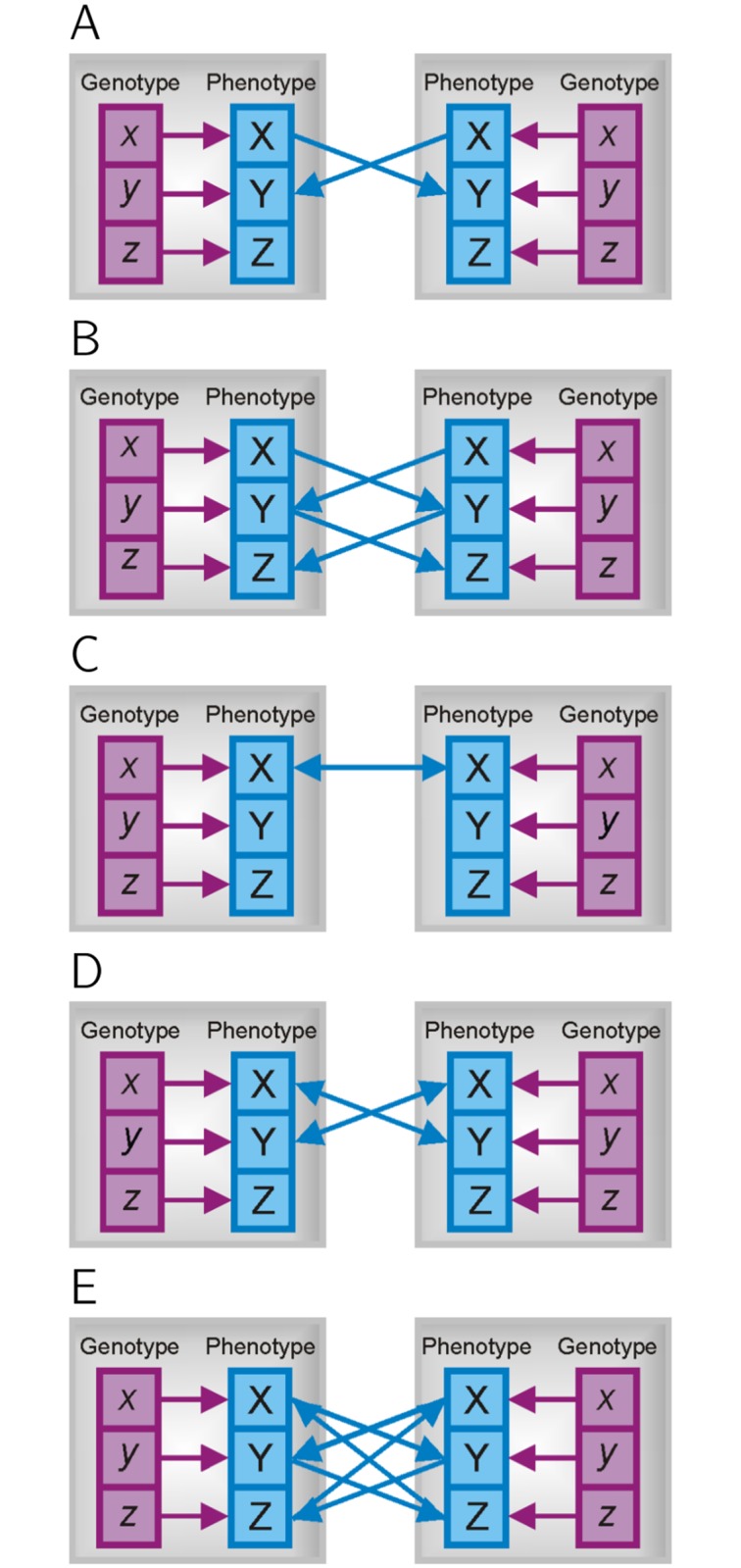
Different classes of interactions. (A) Simple trait interactions. (B) Trait interactions cascades. (C-E) Trait interactions loops. (C) One trait affecting itself. Here, the trait in the focal individual modifies its own expression, in the same individual. (D) Two traits reciprocally affect each other: trait X influences trait Y, which feeds back to trait X. (E) Three traits involved in feedback loop to themselves via multiple interactions. Boxes represent individuals, blue squares represent phenotypic traits, purple squares represent genes. Blue arrows represent matrix Ψ, showing the direction of the influence.

In this model we focus on gradual changes of phenotypes during interactions and investigate repeated, discontinuous interaction events. We assume that in each iteration individuals modify their phenotypes, depending on the phenotypes of others they experienced in the previous encounter. To be consistent with trait-based models, we further assume that interactions are linear and effects additive. Following the distinction made in [37], we investigate three classes of interactions that may occur: simple trait interactions, trait interaction cascades with effects returning to the same individual and trait interactions loops, involving feedback at the trait level (Fig 1).

The first class of simple interactions involves situations in which a trait X in a focal individual influences some other trait Y in its social partners, however, the affected trait does not alter the expression of any other trait, i.e. there is no feedback in the interaction (Fig 1A). An example of such a case is the effect of body size on aggressive behaviour as individuals often mediate their aggressiveness according to the body size of their opponents [38–40].

The second class comprises situations in which a trait in a focal individual (trait X) affects a different trait in its social partners (e.g. trait Y), which in turn influences a third trait Z, but no trait affects itself by any set of partial interactions (Fig 1B). In other words, the effect of the first trait X returns back to the same individual, influencing trait Z. We thus refer to this class as a trait interaction cascade.

The last class describes situations, in which a particular trait affects its own expression through the interaction with other individuals. An example are escalating levels of aggression, where aggressive behaviour of one individual causes aggression in its opponent, which in turn may increase aggressive behaviour of the first individual, as documented, for example, in primate groups [41] (Fig 1C). It is not necessary that only one trait is involved—two distinct traits can reciprocally affect each other, or even more traits can be involved (Fig 1D and 1E). The key point is that the expression of a particular trait feeds back to alter itself, creating an infinite trait interaction feedback loop. In contrast to the above, the feedback is at the trait level.

As many trait-based models pointed out this kind of feedback may drastically affect the expected phenotype [4, 17, 37]. In [37] the authors showed that the equation describing individual phenotypes is not defined for some values of interaction strength, causing extreme phenotypes to occur. It has been shown experimentally that aggressive behaviour can create large differences in reproductive success between individual wasps in a group, and potentially lead to groups breaking up or individuals being evicted [42–46].

In this study, we develop a dynamic model and compare its results to the steady state solutions model in Trubenova and Hager [37]. In our 2012 paper we derived individual phenotypes (trait values) from genotypic values of all participating individuals

$$\begin{array}{ccc} p_{i} & = & \underset{\Gamma^{\prime }}{\underset{︸}{{\left(I+\Psi \right)}^{-1}\left(I+\Psi {\left(I-N\Psi +\Psi \right)}^{-1}\right)}}g_{i}\\ & & +\underset{\Psi^{\prime }}{\underset{︸}{{\left(I+\Psi \right)}^{-1}\Psi {\left(I-N\Psi +\Psi \right)}^{-1}}}\sum_{j\neq i}^{N-1}g_{j} \end{array}$$ (1)

where **g**_**i**_ and **p**_**i**_ are column vectors describing the genotypic and phenotypic values of the *ith* individual, respectively. *N* denotes the number of individuals in the group, *i* the focal individual, *j* denotes social partners and **I** is an identity matrix. The first term **Γ**′ represents direct genetic effects (DGE). The second term represents **Ψ**′, i.e. indirect genetic effects [5, 37]. Matrix **Ψ** is a square interaction matrix [4, 5, 17], in which Ψ_*kl*_ defines the effect of the partner’s trait *l* on the trait *k* of the focal individual. For simplicity we do not consider non-social environment effects in our model, however, these can easily be incorporated into IGE models [4, 5, 17, 37]. All symbols used in the model are described in Table 1.

**Table 1:** Symbols used in the manuscript.

Symbol	Description
***g*** (***p***)	column vector of individual genotypic (phenotypic) values
**Ψ**	square matrix of phenotypic influences; Ψ_*k*, *l*_ denotes the effect of trait *l* on trait *k*
**Γ**′	matrix of direct genetic effects
**Ψ**′	matrix of indirect genetic effects
*N*	number of interacting individuals in the group
$\bar{g}$ $\left(\bar{p}\right)$	column vector of the group mean genotype (phenotype)
Δ ***g***	deviation of an individual’s genotype from mean of its group $\Delta g=g-\bar{g}$
Δ ***p***	deviation of an individual’s phenotype from mean of its group $\Delta p=p-\bar{p}$
*X*, *Y*, *Z*	different traits

## Results

We assume that individuals interact in a series of discontinuous events that modify their phenotypes. For instance, individuals may show altruistic behaviour (e.g. donate food to another individual) according to the amount of help (food) they received in a previous encounter. Rutte and Taborsky [47] showed that cooperative behaviour in female rats is influenced by prior receipt of help, where rats that received help from a social partner were more likely to help their conspecifics. Similarly, recent experiments suggest that this is also the case in humans playing the Prisoner’s Dilemma [48]. In this context, interaction occurs as a series of events (e.g. donating food), that are separated by periods of non-interaction, for instance when individuals are collecting food. The phenotypic trait (amount of donated food) changes discontinuously from one food donation to another.

In such a case, an individual’s propensity to cooperate is given by its genotype, which is independent of interactions. However, the observed phenotypes (i.e. actual amount of food donated) are influenced by the social environment (sum of all phenotypes) that each individual experienced in the previous event (i.e. how much food they received in the previous interaction). For simplicity, we ignore non-social environmental influences.

In the absence of interactions, the phenotype **p_i_** ^(*t*)^ of a focal individual at any given time point (described in arbitrary units) is given by its genotypic values **g_i_**

$$\begin{array}{c} p_{i}^{\left(t\right)}=g_{i}. \end{array}$$ (2)

When individuals interact with social partners, the phenotype of the focal individual after *t* + 1 iterations depends on the phenotypes of conspecifics at the previous time point, after *t* iterations

$$\begin{array}{c} p_{i}^{\left(t+1\right)}=g_{i}+\Psi \sum_{j\neq i}p_{j}^{\left(t\right)}. \end{array}$$ (3)

After *t* repeated iterations the phenotype is given by (see S1 File for derivation)

$$\begin{array}{c} p_{i}^{\left(t\right)}=\sum_{k=0}^{t}{\left(N-1\right)}^{k}\Psi^{k}\bar{g}+\sum_{k=0}^{t}{\left(-\Psi \right)}^{k}\Delta g_{i} \end{array}$$ (4)

where $\bar{g}$ is the mean group genotype and Δ **g**_**i**_ is deviation of the *i* − *th* individual from the mean.

In some cases, individuals may reach phenotypes that no longer change in time, e.g. when after repeated displays of dominance individuals assume their stable positions in a hierarchy. On the other hand, repeated interactions may also escalate the expression of traits such as aggression, potentially leading to group break-up. Whether this happens or not depends on the value of **Ψ**, and the outcomes differ between types of interactions, which we discuss below.

**Simple iterations** In the first scenario, depicted in Fig 1A, trait X is unaffected by interactions and thus remains constant, the trait value given by its genetic value *p*_*Xi*_ = *g*_*Xi*_. However, trait Y depends on trait X expressed in social partners. After a single iteration, the phenotypic value Y in the focal individual is $p_{Yi}=g_{Yi}+\Psi_{YX}\sum_{j\neq i}g_{Xj}$ and remains at this value, independent of any further interaction, because trait X does not change. Thus, phenotypes can be calculated using Eq (1) for any parameter **Ψ**, provided no feedback occurs.

Note that any number of traits may be involved. As long as the affected trait does not influence any other trait, Eq (3) stabilizes after one iteration.

**Trait interaction cascades** The second class comprises interactions with effects returning to the same individual (Fig 1B). Note the difference between this type of interaction and those with trait interaction loops (Fig 1E). In this scenario the affected traits (Y and Z) stabilize after one and two interactions, respectively. If more traits are involved, more iterations may be needed to achieve a stable solution, namely as many as there are steps involved in a given interaction. For instance, in the above case two steps are involved: X influences Y and Y influences Z (Fig 1B). The equilibrium solution is always the same as the one given by Eq (1).

Note that trait Z in the focal individual is not only influenced by the genotypic values of trait Y in other individuals but also by trait X in all individuals, including the focal individual. Thus, the interaction changed the dependence of the focal individual’s phenotype on its own genotype, i.e. the direct genetic effect (DGE).

**Trait interaction loops** In the third, most complicated scenario, a trait affects its own expression either directly (Fig 1C) or via interactions with different traits (Fig 1D and 1E). For example, aggressive behaviour may induce an aggressive response in social partners, or, in more complicated scenarios, trait X affects trait Y, which affects a third trait Z, and this trait Z affects again the expression of trait X. Unlike in the previous class, here the feedback is to the same trait, creating an infinite feedback loop.

For example, consider just one trait X that affects expression of the same trait in social partners. In univariate form Eq (3) simplifies to

$$\begin{array}{c} p_{i}=\sum_{k=0}^{t}{\left(N-1\right)}^{k}\Psi^{k}\bar{g}+\sum_{k=0}^{t}{\left(-\Psi \right)}^{k}\Delta g_{i}. \end{array}$$ (5)

Whether the trait values stabilize after some number of repeated interactions (meaning that any following interactions do not significantly change phenotypes) now depends on the parameter Ψ. Both parts of Eq (4) must converge because $\bar{g}$ and Δ *g_i_* are independent. The first part $\sum_{k=0}^{t}{\left(N-1\right)}^{k}\Psi^{k}\bar{g}$ converges for *t* → ∞ (an increasing number of iterations) within the range defined by $\left(\frac{-1}{N-1},\frac{1}{N-1}\right)$ and the second part $\sum_{k=0}^{t}(\Psi^{2k}-\Psi^{2k+1})\Delta g_{i}$ converges within (−1, 1). Therefore, Eq (4) leads to a stable solution only for Ψ within the interval $\left(\frac{-1}{N-1},\frac{1}{N-1}\right)$ and this solution is the same as the one given by Eq (1).

Phenotypes cannot stabilize if Ψ is outside this interval. If individuals have phenotypes described by Eq (1) these remain unchanged in the absence of other influences. However, even a small deviation, for example the arrival of a new group member, forces phenotypes to diverge from this solution, which means that the solution represents an unstable equilibrium point. Thus, it is unlikely that individuals have phenotypes predicted by Eq (1) when Ψ lies outside of the convergence interval.

**Fig 2:**
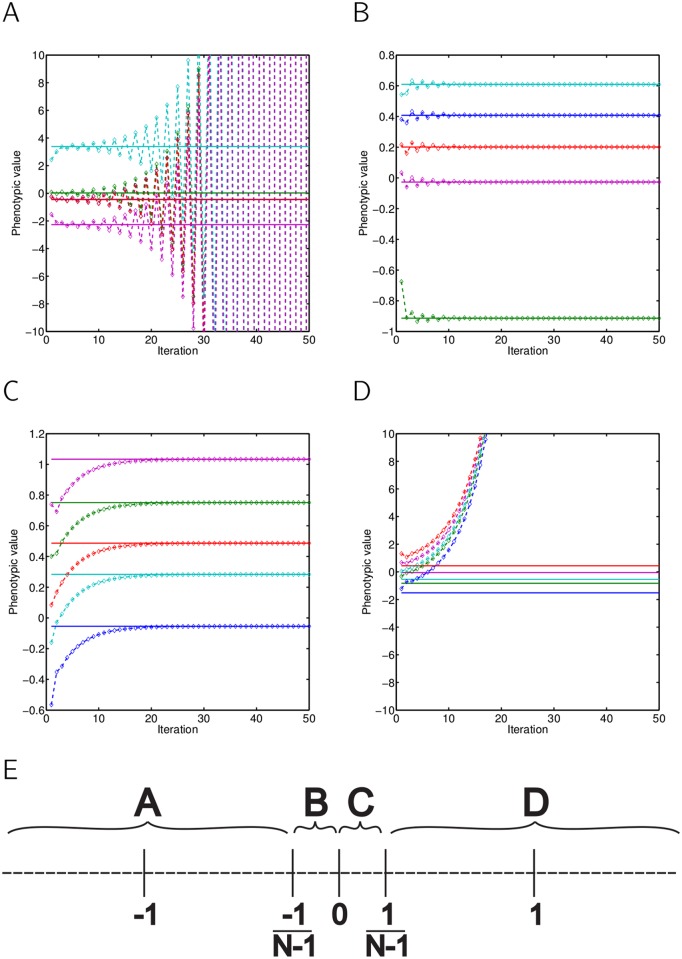
Four possible types of dynamics when a given trait affects its own expression in social partners (univariate scenario, Fig 1C). (A) For strong negative feedback, phenotypes of individuals oscillate with increasing magnitude. (B) For weak negative feedback, phenotypes oscillate with decreasing magnitude and converge to values given by Eq (1). (C) Phenotypes monotonically converge to the stable solution, if weak positive feedback occurs. Solid lines represent phenotypes given by Eq (1), diamonds represent phenotypes of individuals after simulated iterations of Eq (3). Different colors represent different individuals. Agent-based simulations of five interacting individuals. (D) but diverge in the same direction, if positive feedback is too strong. (E) The strength of interaction Ψ determines the dynamics of phenotypic changes. A-D letters correspond to panels (A-D).

Fig 2 shows simulations of phenotypes of five individuals changing in time for different values of Ψ, when one trait affects its own expression. In our simulations, individuals have genotypic values randomly assigned to them from the uniform distribution, and at the beginning of interaction their phenotypes are given just by their genotypes, using (Eq 2). In each following step (iteration), their phenotypes are adjusted according to the phenotypes experienced in the previous encounter, using (Eq 3). See S2 File for detailed information about the simulations. Note the different dynamics that can occur due to the interaction: strong negative feedback will lead to phenotypes oscillating with an increasing magnitude (Fig 2A), while weak negative interactions ($-\frac{1}{N-1}<\Psi <0$) lead to phenotypes oscillating with decreasing magnitude (Fig 2B). In case of positive but weak interactions ($0<\Psi <\frac{1}{N-1}$) phenotypes will converge to the equilibrium point monotonically (Fig 2C). For example, if aggression weakly enhances aggressive behaviour of social partners, all individuals will become slightly more aggressive, but soon reach a stable level (Fig 2C). However, if displays of aggression reduce aggression in others, aggressive behaviour of a particular individual will oscillate before reaching a stable level (Fig 2B). When there is strong positive feedback ($\frac{1}{N-1}<\Psi$, meaning that the total influence of the social partner phenotypes is larger than 1), phenotypes diverge in the same direction (Fig 2D); e.g. aggressive behaviour escalates until the group breaks up. Fig 2E shows the dependence of dynamics on interaction strength Ψ.

This is in line with game theory models developed by André and Day [31] who investigated the iterated prisoner's dilemma and demonstrated that the degree of cooperation depended on the partner's responsiveness. When responsiveness was weak (corresponding to small values of Ψ in our model), the degree of cooperation converged to a fixed point. By contrast, greater responsiveness led to increased errors and thus stronger reactions to a partner's move.

**Fig 3:**
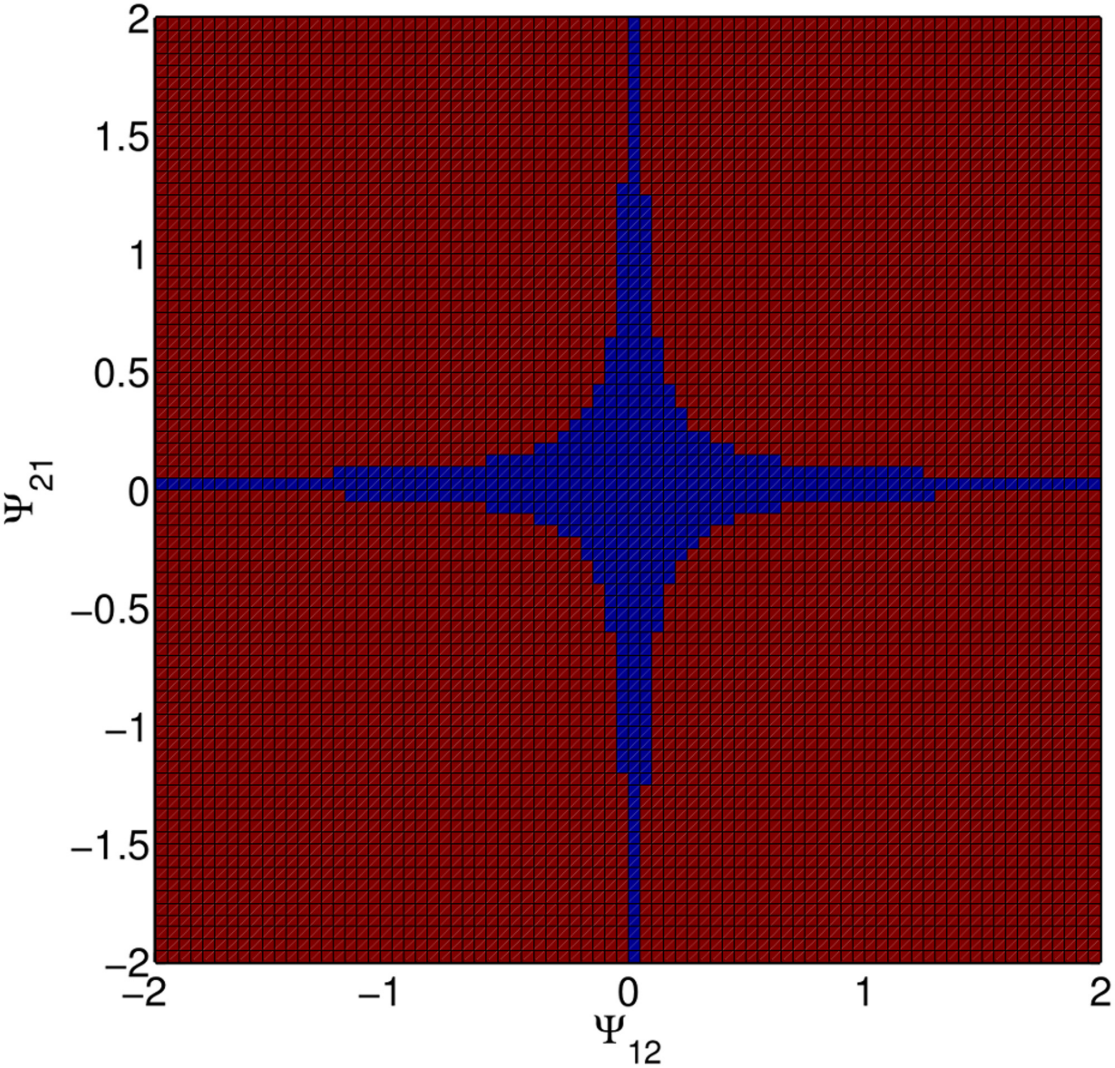
Multi-trait scenario, when two traits reciprocally influence each other (Fig 1D). Here, the strength of interaction determines whether phenotypes stabilize at phenotypic values given by Eq (1) (blue), or diverge, causing extreme phenotypes (red). Due to the presence of the feedback loop, only weak interactions will lead to stable phenotypes. Generated from agent-based simulations of five interacting individuals.

Generalizing the single trait example to multivariate scenarios, for instance when multiple traits are involved in feedback loops (Fig 1D and 1E), is straightforward. The sufficient condition for the convergence of the multivariate expression (Eq 4) is that all eigenvalues of matrix (*N* − 1)**Ψ** are smaller than 1 (see S1 File for details). Note that even if this condition is violated, expression (Eq 1) is a steady state solution, however an unstable one. Fig 3 shows areas where phenotypes diverge (red) or converge to the solution given by Eq (1) (blue), when two traits affect each other (Fig 1D).

## Discussion

In this study, we analysed iterative interactions among individuals and their effect on social phenotypes. Our analysis highlights several potential limitations in the applicability of some IGE model predictions based on unrealistic assumptions about the nature of interactions in biological systems.

We showed that when there is no feedback loop, iterative interactions lead to a stable solution for any interaction coefficient **Ψ**, which is the same as in Trubenova and Hager [37]. However, in cases where one trait affects its own expression via interactions with conspecifics (as in Fig 1C–1E) iterative interactions between individuals yield only a limited parameter range in which stable solutions are reached. Within this interval phenotypes stabilize at the steady state point given by Eq (1), independent of initial phenotypes. However, outside this interval the equilibrium solution inferred by Trubenova and Hager [37] describes an unstable fixed point. As a consequence, certain trait values predicted by models are unstable or transitions between states may never occur in a given interaction. To date the nature and consequences of these limitations have not been formally explored. For instance, in Moore et al. [4] all trait values are standardised and hence the interaction coefficient Ψ is limited to values between −1 and 1. However, this range seems to apply to dyadic interactions only. In the univariate scenario of their multimember model, McGlothlin et al. [17] restricted the parameter space to between −1 and 1/(*N* − 1), stating that at these values the denominator in their phenotypic equation equals zero. However, this appears more of a mathematical limitation of the model rather than reflecting biological scenarios.

There are two possible ways to interpret unstable points. First, if individuals have phenotypes given by Eq (1) these will remain unchanged only if not perturbed. However, in real biological systems it is rather more likely that changes in the social environment occur, which may cause such perturbations and thus lead to more extreme phenotypes. This phenomenon can be illustrated using the example of aggression. If the relative effect of aggressive behaviour in social partners (*N* − 1)Ψ is smaller than 1, repeated interactions may lead to a level of aggression that is stable. If, however, a new group member arrives and the relative effect of aggressive behaviour (*N* − 1)Ψ is larger than 1, expressions of aggression escalate to a degree that is no longer compatible with group stability. Thus, the interaction coefficient **Ψ** may place constraints on the maximum predicted stable group size.

Furthermore, our results suggest that if phenotypes are not stable but oscillate, evolutionary outcomes will strongly depend on the properties (i.e. the period and magnitude) of oscillations, as well as the time point when selection occurs because the selectable phenotypes change considerably. This may enhance stochasticity of selective processes. Thus, IGEs may actually enhance an element of stochasticity in evolution.

The second interpretation considers unstable points to be artefacts of the linear IGE approach. Most IGE models assume additive effects of conspecific phenotypes on the focal individual. Further, it is assumed that **Ψ** is independent of the number of interacting individuals *N* (with few exemptions, e.g. [10, 13]). However, these assumptions are almost certainly violated in large groups as particular individuals are less likely to interact, or in cases where strong feedback loops at the level of traits occur. It is possible that the effect of individuals on the phenotypes of conspecifics is linear in some range (and in first two classes of interactions individuals stay in this range). Yet, the feedback loops in the third class of interactions may cause the departure of the system from this linear range, when interaction effects grow too strong. Therefore, while the linear model may be a reasonable approximation of biologically relevant scenarios in the first two classes, it may not sufficiently describe situations when feedback loops at the trait level occur. Maternal provisioning may serve to illustrate this point. Provisioning of offspring by their mother may initially linearly depend on the level of begging by offspring [49–51]. If provisioning does not influence begging behaviour, the relationship between the two may be linear in some range—more offspring or offspring begging more aggressively may solicit overall more food from their mothers. However, if increased provisioning strongly encourages elevated begging behaviour, it is unlikely that the mother can always increase her provisioning accordingly. It is more likely that at some point, even if offspring beg more, she is not able to increase the rate of provisioning [52]. Thus, in such a case, it may be necessary to add a term describing the carrying capacity of the mother for provisioning into the model. It is possible that additive, linear models do not sufficiently describe interactions when feedback loops occur, and non-linear terms may have to be included in the model.

Another approach to modelling social interactions would be to investigate the nature of these interactions, i.e. how exactly do individuals influence each other. Are these interactions continuous, or do they occur in distinct events? How quickly do phenotypes adjust to the change in their social environment, and how does this compare to generation time? Does modelling such interactions lead to phenotypes that stabilize after a while? Only if we find a stable solution and if the phenotypic change is sufficiently faster than generation time, we can ignore the dynamics of interactions and use equations for stable equilibrium.

To avoid using solutions that are not biologically feasible, the approach developed by Roughgarden, Akçay and Van Cleve [32, 33] may offer a solution. This two-tiered framework firstly focuses on social interactions at the behavioural time scale, finding stable phenotypes resulting from the interactions. Only then the evolutionary dynamics are considered. This modelling approach is more complicated than traditional trait-based IGE approach, and often may not be treated analytically. However, more complex scenarios could be simulated by agent-based models, and ultimately may be better for representing real biological problems than simplified analytical models.

## Supporting Information

S1 File Mathematical inference of the results. (PDF)

S1 Fig Comparison of three methods of phenotype calculation. The solution calculated using Equation (S9) (crosses) agrees with the one obtained by iterating Eq (S1) (circles), and may (A) or may not (B) converge to the stable state solution calculated by Eq (1) (solid line). Two traits reciprocally influence each other (X and Y) in five interacting individuals. (A) Ψ_12_ = Ψ_21_ = 0.2. (B) Ψ_12_ = Ψ_21_ = 0.5. Different colors represent different individuals.
(PDF)

S2 File Description of the agent based simulations. (PDF)
