# Supplementary material for: Indirect Genetic Effects and the Dynamics of Social Interactions
Source: PLoS One. 2015 May 18;10(5):e0126907. doi: 10.1371/journal.pone.0126907 (PMC4436347; doi:10.1371/journal.pone.0126907)
Supplement: S1 File — (PDF) [file pone.0126907.s001.pdf]

# S1 File: Mathematical inference

For simplicity, we omitted matrix $\mathbf{\Gamma}$ when analysing the dynamics of interactions, assuming that $\boldsymbol{g}$ is now a vector of genotypic values, not genotypes, the same as $\mathbf{\Gamma}\boldsymbol{g}$ in [1].

When individuals interact with social partners, the phenotype of the focal individual at time $t+1$ depends on the phenotypes of conspecifics at time $t$ as

$$\begin{aligned} \boldsymbol{p_i}^{(t+1)} &= \boldsymbol{g_i} + \boldsymbol{\Psi} \sum_{\boldsymbol{j \neq i}} \boldsymbol{p_j^{(t)}} \\ &= \boldsymbol{g_i} + \boldsymbol{\Psi N \overline{p}^t} - \boldsymbol{\Psi p_i^t}. \end{aligned} \tag{S1}$$

We want to express each phenotype after $n$ iterations as a function of the genotypes of social partners. The phenotype of the focal individual before interaction is

$$\boldsymbol{p_i}^{(0)} = \boldsymbol{g_i} \tag{S2}$$

and the mean group phenotype before interaction

$$\boldsymbol{\overline{p}}^{(0)} = \boldsymbol{\overline{g}} \tag{S3}$$

where $\overline{\mathbf{g}} = \sum_j \boldsymbol{g_j}/N$ ($\overline{\mathbf{p}} = \sum_j \boldsymbol{p_j}/N$) is the mean genotype (phenotype).

The phenotype of the focal individual after one iteration is given by

$$\boldsymbol{p_i}^{(1)} = \boldsymbol{g_i} + \boldsymbol{\Psi N \overline{p}^0} - \boldsymbol{\Psi p_i^0} \tag{S4}$$

$$= \boldsymbol{g_i} + \boldsymbol{\Psi N \overline{g}^0} - \boldsymbol{\Psi g_i^0}. \tag{S5}$$

From (S4) we can express the mean group phenotype after one iteration

$$\boldsymbol{\overline{p}}^{(1)} = \boldsymbol{(I - \Psi} + N\boldsymbol{\Psi)\overline{g}}. \tag{S6}$$

Again, substituting (S6) to (S1), we can express the phenotype of the focal individual after two iterations as

$$\begin{aligned} \boldsymbol{p_i}^{(2)} &= (I - \Psi + \Psi\Psi)\boldsymbol{g_i} + (N\boldsymbol{\Psi}(\boldsymbol{I} - \boldsymbol{\Psi}) + N^2\boldsymbol{\Psi}\boldsymbol{\Psi} - N\boldsymbol{\Psi}\boldsymbol{\Psi})\bar{\boldsymbol{g}} \\ &= (I - \Psi + \Psi\Psi)(\bar{\boldsymbol{g}} + \triangle\, \boldsymbol{g_i}) + (N\boldsymbol{\Psi}(\boldsymbol{I} - \boldsymbol{\Psi}) + N^2\boldsymbol{\Psi}\boldsymbol{\Psi} - N\boldsymbol{\Psi}\boldsymbol{\Psi})\bar{\boldsymbol{g}} \end{aligned} \quad \text{(S7)}$$

which can be written as

$$\boldsymbol{p_i}^{(2)} = \sum_{k=0}^{2}(N-1)^k\boldsymbol{\Psi}^k\bar{\boldsymbol{g}} + \sum_{k=0}^{2}(-\boldsymbol{\Psi})^k \triangle\, \boldsymbol{g_i} \quad \text{(S8)}$$

where $\triangle\, \mathbf{g_i} = \boldsymbol{g_i} - \bar{\boldsymbol{g}}$ (also $\triangle\, \mathbf{p_i} = \boldsymbol{p_i} - \bar{\boldsymbol{p}}$) is deviation of the $i$-th genotype (phenotype) from the mean.

By induction, we conclude that the individual's phenotype after $n$ generations is given by

$$\boldsymbol{p_i}^{(n)} = \sum_{k=0}^{n}(N-1)^k\boldsymbol{\Psi}^k\bar{\boldsymbol{g}} + \sum_{k=0}^{n}(-\boldsymbol{\Psi})^k \triangle\, \boldsymbol{g_i}\ . \quad \text{(S9)}$$

Fig. A shows phenotypes calculated in three different ways: iterating equation (S1), using equation (S9) or phenotypic equation inferred by [1] for phenotypes at the equilibrium point. Clearly, trajectories created from equations (S1) and (S9) agree, but they may or may not converge to the stable state solution given by equation (1).

For $n \to \infty$, equation (S9) converges if both of its summands converge. This is the case if both matrices $(N-1)\boldsymbol{\Psi}$ and $(-\boldsymbol{\Psi})$ have a spectral radius $\rho$ of less than one, i.e., all their eigenvalues $\lambda$ fulfil $|\lambda| < 1$. Since for $N > 1$ $\rho[(N-1)\boldsymbol{\Psi}] > \rho(\boldsymbol{\Psi}) = \rho(-\boldsymbol{\Psi})$, our sufficient condition for the convergence of equation (S9) is $\rho[(N-1)\boldsymbol{\Psi}] < 1$. In this case, we obtain

$$\begin{aligned} \boldsymbol{p_i}^{(\infty)} &= \sum_{k=0}^{\infty}(N-1)^k\boldsymbol{\Psi}^k\bar{\boldsymbol{g}} + \sum_{k=0}^{\infty}(-\boldsymbol{\Psi})^k \triangle\, \boldsymbol{g_i}\ . \\ &= (\mathbf{I} - (N-1)\boldsymbol{\Psi})^{-1}\bar{\boldsymbol{g}} + (\mathbf{I} + \boldsymbol{\Psi})\Delta\boldsymbol{g_i}. \end{aligned} \quad \text{(S10)}$$

This solution is equivalent to equation (1), as can be seen after substituting for $\Delta \boldsymbol{g_i}$ and $\bar{\boldsymbol{g}}$ accordingly. Note that even if $\rho[(N-1)\boldsymbol{\Psi}] < 1$ is violated, expression (S10) is a steady state solution of recursion (S1), however an unstable one.

# References

[1] Trubenová B, Hager R (2012) Phenotypic and evolutionary consequences of social behaviours: Interactions among individuals affect direct genetic effects. PLoS ONE 7: e46273.

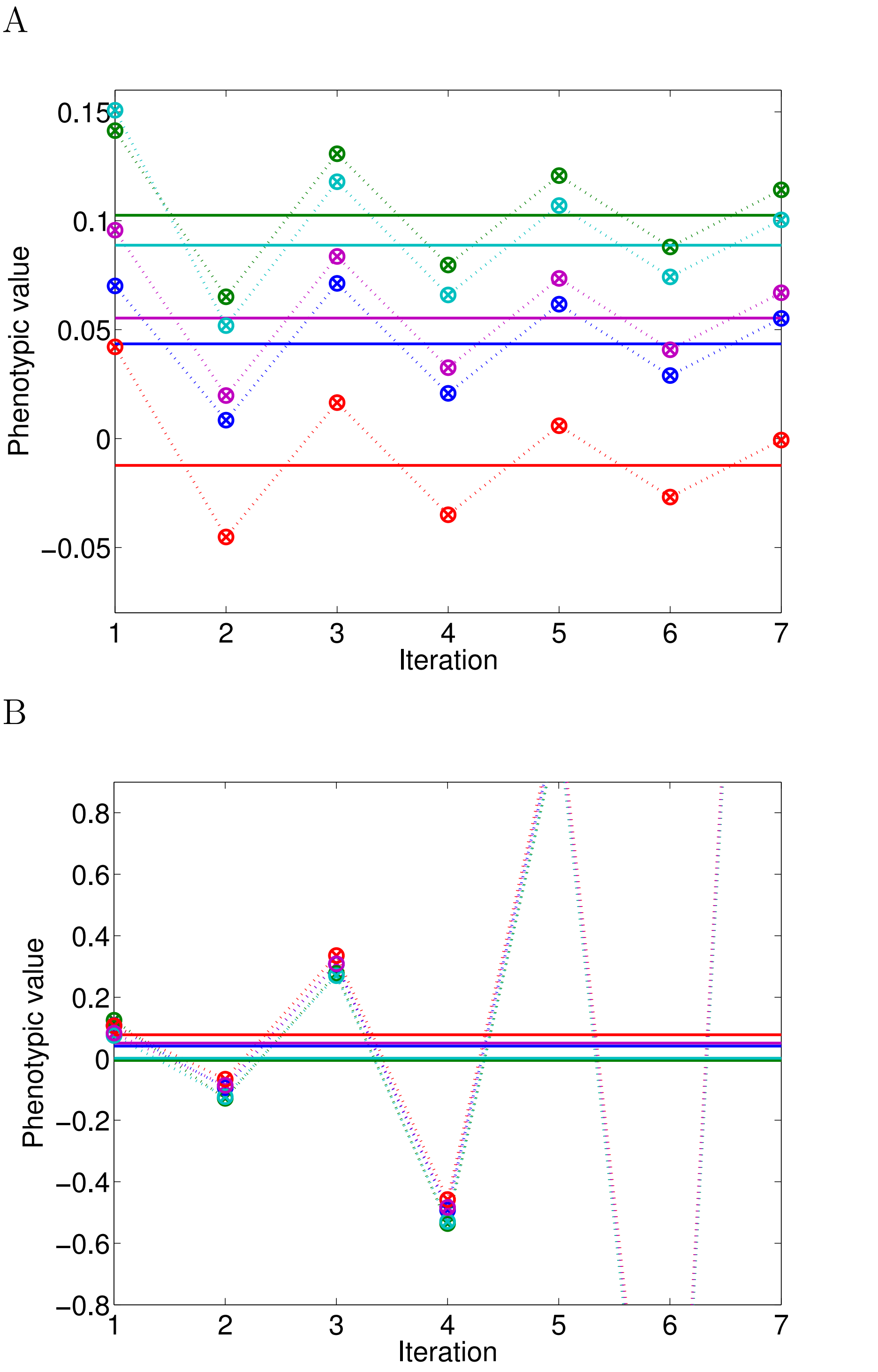


Fig. A: **Comparison of three methods of phenotype calculation.** The solution calculated using equation (S9) (crosses) agrees with the one obtained by iterating equation (S1) (circles), and may (A) or may not (B) converge to the stable state solution calculated by equation (1) (solid line). Two traits reciprocally influence each other (X and Y) in five interacting individuals. (A) $\Psi_{12} = \Psi_{21} = 0.2$. (B) $\Psi_{12} = \Psi_{21} = 0.5$. Different colors represent different individuals.
