# Supplementary figures and images for: Indirect Genetic Effects and the Dynamics of Social Interactions
Source: PLoS One. 2015 May 18;10(5):e0126907. doi: 10.1371/journal.pone.0126907 (PMC4436347; doi:10.1371/journal.pone.0126907)

A

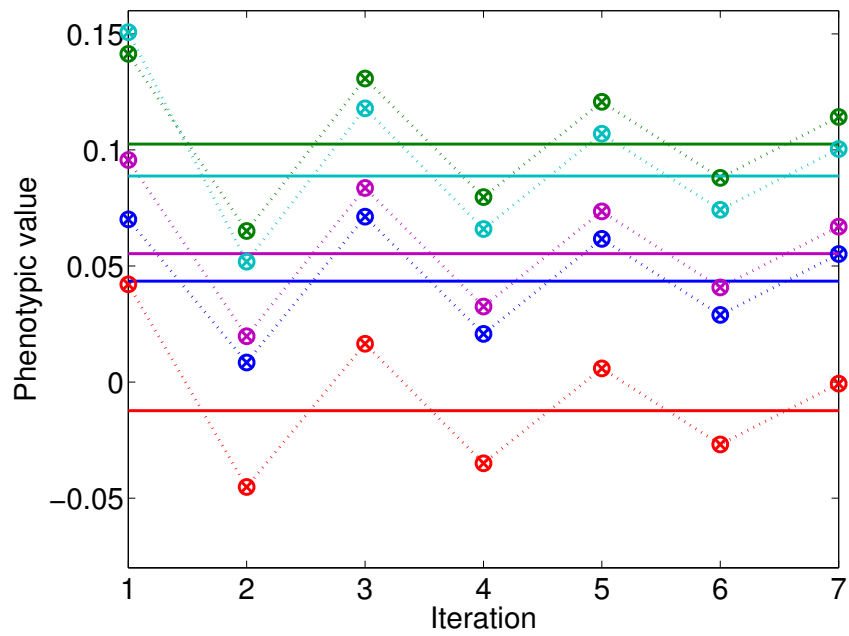

B

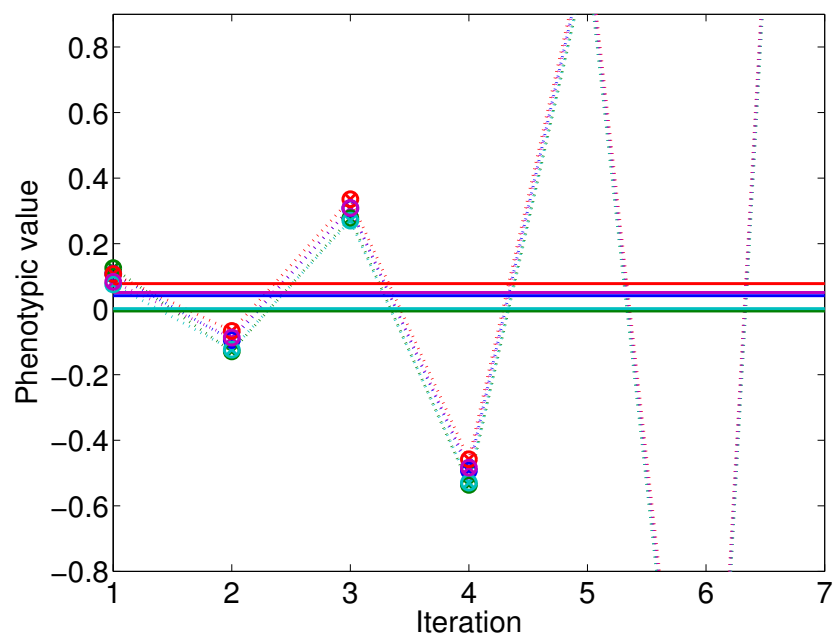

Figure S1

Supplement: S1 Fig — The solution calculated using Equation (S9) (crosses) agrees with the one obtained by iterating Eq (S1) (circles), and may (A) or may not (B) converge to the stable state solution calculated by Eq (1) (solid line). Two traits reciprocally influence each other (X and Y) in five interacting individuals. (A) Ψ12 = Ψ21 = 0.2. (B) Ψ12 = Ψ21 = 0.5. Different colors represent different individuals. (PDF) [file pone.0126907.s002.pdf]
