# Supplementary material for: Indirect Genetic Effects and the Dynamics of Social Interactions
Source: PLoS One. 2015 May 18;10(5):e0126907. doi: 10.1371/journal.pone.0126907 (PMC4436347; doi:10.1371/journal.pone.0126907)
Supplement: S2 File — (PDF) [file pone.0126907.s003.pdf]

S2 File: Simulations

To support our results, we simulated interactions among individuals. Each individual is defined by two vectors, one genotypic and one phenotypic, each with three components (we assume that each individual has three phenotypic traits and three genes). At the beginning of the simulation, genotypic values are assigned randomly from a uniform distribution and do not change during the simulation. Phenotypic vectors are initialized as being equal to genotypic vectors.

We systematically varied the strength of interaction $\boldsymbol{\Psi}$ in various scenarios (class 1-3), and simulated 500 iterations of interactions for each parameter set. In each round, the mean phenotype was calculated and all individuals adjusted their phenotypes following equation (S1). At the end of the simulations, the distance between simulated individuals phenotypes and phenotypes determined using equation (1) was calculated. If this distance was less than 10^{-5}, phenotypes were assumed to be converging to the solution given by equation (1).

The univariate scenario, when only one trait affects its own expression (Fig. 1 C and 2), is simulated by defining $\boldsymbol{\Psi} = \begin{bmatrix} \Psi & 0 \\ 0 & 0 \end{bmatrix}$. The multivariate scenario, with two traits reciprocally affecting each other (Fig. 1D, 2 and S1), is simulated by defining $\boldsymbol{\Psi} = \begin{bmatrix} 0 & \Psi_{21} \\ \Psi_{12} & 0 \end{bmatrix}$.
